# The role of self-regulatory skills and automaticity on the effectiveness of a brief weight loss habit-based intervention: secondary analysis of the 10 top tips randomised trial

**DOI:** 10.1186/s12966-017-0578-8

**Published:** 2017-09-05

**Authors:** Nathalie Kliemann, Victoria Vickerstaff, Helen Croker, Fiona Johnson, Irwin Nazareth, Rebecca J. Beeken

**Affiliations:** 1 Department of Behavioural Science & Health, University College London, London, England; 2 Department of Primary Care & Population Health, University College London, London, England; 3 Leeds Institute of Health Sciences, University of Leeds, Level 10, Worsley Building, Clarendon Way, Leeds, LS2 9NL England

**Keywords:** Self-regulation, Habit formation, Automaticity, Weight loss, Intervention

## Abstract

**Background:**

Habit-interventions are designed to promote the automaticity of healthy behaviours and may also enhance self-regulatory skills during the habit-formation process. A recent trial of habit-based advice for weight loss (10 Top Tips; 10TT), found that patients allocated to 10TT lost significantly more weight over 3 months than those allocated to usual care, and reported greater increases in automaticity for the target behaviours. The current study aimed to test the hypothesis that i) 10TT increased self-regulatory skills more than usual care, and ii) that self-regulatory skills and automaticity changes mediated the effect of 10TT on weight loss.

**Methods:**

537 obese patients from 14 primary care practices in the UK were randomized to receive 10TT or usual care. Patients in the 10TT group received a leaflet containing tips for weight loss and healthy habits formation, a self-monitoring log book and a wallet-sized shopping guide on how to read food labels. Patients were weighed and completed validated questionnaires for self-regulation and automaticity at baseline and 3-month follow-up. Within-group and Between-group effects were explored using Paired T-test and ANCOVA, respectively. Mediation was assessed using bootstrapping to estimate indirect effects and the sobel test.

**Results:**

Over 3 months patients who were given 10TT reported greater increases in self-regulatory skills (Mean difference: .08; 95% CI .01; .15) than those who received usual care. Changes in self-regulatory skills and automaticity over 3 months mediated the effect of the intervention on weight loss (β = .52, 95% Bias Corrected CI .17; .91).

**Conclusions:**

As hypothesised, 10TT enhanced self-regulatory skills and changes in self-regulatory skills and automaticity mediated the effect of the intervention on weight loss. This supports the proposition that self-regulatory training and habit formation are important features of weight loss interventions.

**Trial registration:**

This study was prospectively registered with the International Standard Randomised Controlled Trials (ISRCTN16347068) on 26 September 2011.

**Electronic supplementary material:**

The online version of this article (10.1186/s12966-017-0578-8) contains supplementary material, which is available to authorized users.

## Background

Obesity is a growing public health concern that affects more than 600 million adults worldwide [1, 2] and increases risk for chronic diseases [2, 3]. There is therefore a need to find effective interventions to help individuals with obesity to lose weight and to prevent weight gain at the population level. While commercial programmes are promising, they require a certain level of motivation on the part of the individual, such as attendance at group sessions over a prolonged period of time.

Evidence from recent studies suggests that brief habit-based interventions [4, 5], may be an innovative approach for promoting weight loss, even when conscious motivation is low [5]. Habit-based interventions promote the repetition of target behaviours in a consistent context in order to make them become more automatic and habitual [6, 7]. Habits are formed through learned associations between a cue or stimulus with a response, so that when a cue is encountered it automatically generates an impulse toward action [8]. Although interest is growing in habit-formation approaches [5, 7, 9], weight loss interventions applying this approach are still scarce [10, 11] and their mechanisms of action are not completely understood. Improving the theoretical understanding of how habit-based interventions bring about weight loss is pressing, as it may provide guidance on the development of more effective interventions [12] to tackle the obesity epidemic.

A recent habit-based weight loss intervention, called 10 Top Tips (10TT), was developed as a leaflet to promote a set of everyday healthy eating and activity behaviours [10] in obese patients (*n* = 537) from primary care in the UK [4]. The advice for turning these target behaviours into habits involved the recommendation of making specific plans and repeating the behaviours in a consistent context, as well as monitoring performance daily using a log book. The active treatment was defined as the first three months [6], which is the period usually required to form habits [9, 13]. The results of this trial demonstrated that over three months patients allocated to 10TT lost 0.87 kg (95% CI -1.47; −.027, adjusted mean) more than those allocated to usual care. Furthermore, patients who received 10TT reported a greater increase in automaticity of the target behaviours (adjusted sum difference = 8.45, 95% CI =2.59, 14.32) over three months, which suggests that 10TT was more effective at establishing new habits by the end of the intervention period. However, whether this increase in automaticity was the mechanism behind the observed weight loss remains to be explored.

The process of habit formation may also require self-regulatory skills to translate the intended behaviour into action and override unwanted automated responses [9, 14]. Self-regulation refers broadly to the multiple processes involved in goal-directed behaviour [15] and encompasses the ability to alter behaviour, thoughts, feelings, attention and environment in the pursuit of personal goals [16–19]. The capacity to self-regulate behaviour is considered to be a relatively stable construct [20], but one that can be improved through practice [21, 22]. Since, the habit acquisition phase within 10TT involves actions such as setting goals, planning, self-monitoring and reviewing progress [23], it is possible that it may also enhance self-regulatory skills. Recent studies have suggested that self-regulatory skills may be an important individual factor that helps individuals to achieve, as well as maintain a healthy weight and diet [21, 24, 25]. Examining the impact of the 10TT intervention on self-regulatory skills may deepen our understanding of the mechanisms underlying the effectiveness of habit-based weight loss interventions.

Therefore, the current study aimed to explore the mechanisms behind the weight loss observed in the 10TT trial. Specifically, this study aimed to investigate the effect of 10TT on self-regulatory skills and whether changes in self-regulatory skills and automaticity mediated the effect of the intervention on weight loss over three months. Data from returned 10TT log books were also explored to provide an indication of engagement with the intervention, and how this related to changes in self-regulatory skills, automaticity and weight loss. We hypothesised that i) 10TT increased self-regulatory skills more than usual care, ii) that self-regulatory skills and automaticity changes mediated the effect of 10TT on weight loss and iii) that patients with the greatest improvement in self-regulatory skills, automaticity and weight would be more engaged in terms of their use of the log books.

## Methods

### Study design

This study is a secondary analysis from a two-arm, individually-randomised (1:1 ratio), controlled trial in obese adults in primary care, comparing the 10TT intervention with ‘usual care’. The active treatment was defined as the first three months. The protocol of the trial has been published elsewhere [6]. Ethical approval was granted by South East London Research Ethics Committee 2 via IRAS (Ref No. 10/H0802/59, Approval granted 9th July 2010). The trial was conducted in accordance with the CONSORT 2010 guidelines (see Additional file 1).

### Participants and recruitment

Participants were patients from General Practices in England, who were classified as obese (BMI ≥ 30) and were 18 years or older. Patients were excluded if they were pregnant; terminally ill; or unable to provide informed consent due to mental incapacity or active psychotic illness. A total of 14 General Practices across England were selected through the General Practice Research Framework, to represent socio-economically deprived and ethnic diverse populations; and both urban and rural areas. Detailed information about the 14 practices included in this study has been published elsewhere [4, 6]. All obese patients registered in these practices were invited to take part in the study from August 2010 to October 2011. However, when the number of patients with obesity registered in the practice exceeded 500, a random sample of 500 was selected and invited. The practices sent a letter to eligible participants including the information sheet and an ‘expression of interest’ form. Potential participants met with a health professional, who checked their eligibility, explained the study and took informed consent.

### Randomisation

Randomisation took place after eligibility was confirmed and after completion of baseline measures. It was done by telephoning a central randomisation service (Health Service Research Unit at Aberdeen) to ensure allocation concealment. A computer-based list generated random permuted blocks of size 2 to 4. The randomisation was stratified by practice in order to have a socio-economic balance between the groups.

### 10TT

Participants randomised to 10TT received the 10TT leaflet, a self-monitoring log book and a wallet sized shopping guide on how to read food labels. These were provided to patients at their baseline appointment by a trained health professional (nurses or health care assistants), who spent about 30 min talking through the leaflet with the patients, following a standardized script. After providing the patients with the materials and information about the intervention, there was no further clinical contact. Participants could request more log books when necessary and were instructed to return the completed ones by post in a provided free-post envelope. Further information about the intervention content has been previously published [6].

### Usual care

Patients randomised to usual care received the practice’s usual care, which consisted of referring patients to community programmes (12 weekly sessions) or to a health professional not involved in the trial for a discussion on healthy eating (usually at least 2 appointments). Detailed information about the usual care received by participants has been previously published [4].

### Blinding

Participants were not blinded to their group condition. However, the assessment at 3 months was done by health professionals blinded to participant condition allocation.

### Measures

#### Demographic characteristics

Socio-demographic characteristics, including gender, age, ethnic origin and education, were obtained from health records.

#### Anthropometric measures

Body weight (in kg) was measured using TANITA scales supplied to the practices for use only in this study and height (in cm) was taken using the Practice equipment. Weight changes from baseline to 3-month follow-up were calculated.

#### Behavioural measures

Self-regulatory skills were assessed using the validated 31-item Self-Regulation Questionnaire- SRQ [26], adapted for eating and weight self-regulatory skills. The adaptation consisted of changing the wording to make the items apply specifically to weight and dietary self-regulation. For example, ‘I’m able to accomplish goals I set for myself’ was changed to ‘I’m able to accomplish weight loss goals I set for myself’. The Likert response scale was changed from 5 to 4 options by removing the ‘uncertain or unsure’ option. The scores ranged from 1 (strongly disagree) to 4 (strongly agree). As the original scale has only one factor [26], the outcome for self-regulatory skills was the mean score for the 31 items. Baseline data for the adapted questionnaire showed it had good internal reliability (Cronbach’s alpha of .88). The mean score for the 31 items and the mean change from baseline to 3 month follow-up were calculated.

The automaticity of the 10 targeted eating and activity behaviours plus self-weighing behaviour was assessed using a single item taken from the 12-item Self-Report Habit Index [27], asking how much each of the behaviours were done automatically on a 7-point Likert scale from ‘none of the time’ to ‘all of the time’. The scores ranged from 1 to 7. For some of the target behaviours more than one question was generated to better assess the automaticity of the behaviour. For example, for ‘Focus on your food’ behaviour, two questions on how automatically people eat in front of the TV and at a table were generated (see Additional file 2: Table S2). The mean score for the automaticity of all 16 behaviours was calculated as well as the mean change from baseline to 3-month follow-up for each behaviour (see Additional file 3: Table S3).

#### 10TT log books

The log books had tick sheets, where participants could record whether they managed each tip and record their weight every day. They also contained notes and planning sheets, where participants could write down how they aimed to achieve each tip every week. Data from the 10TT log books returned at 3 months were: the number of weeks the self-monitoring, weight recording and planning sections were used, the number of times the target behaviours were managed at least 5 times per week and the average number of tips managed per week. The use of the 10TT log books in relation to the level of change in self-regulatory skills, automaticity and weight over 3 months were explored using ranked percentiles: percentile <75 represented medium to small changes and percentile ≥75 represented large changes.

### Sample size

The trial was powered to detect a significant weight difference between the intervention and control group as published previously [6]. Therefore, the analyses presented in this study should be considered exploratory.

### Statistical analyses

Data were analysed using SPSS version 22.0 (SPSS Inc). All analyses were according to intention-to-treat. Initially a completer analysis was performed using complete data at baseline and follow-up for each outcome. Participants with more than 20% of missing data for the self-regulation and automaticity questionnaires were excluded from the completer analysis. When there were up to 20% missing data for these questionnaires the individual median score was imputed. Baseline descriptive analyses were applied. To explore baseline differences between the completers and non-completers at 3 months, chi-square tests for categorical variables and t-tests for continuous variables were applied.

Following the trial’s analysis plan [6], we checked for clustering by GP practice by running unconstrained models in the mixed effect models and calculating the intracluster correlation coefficients (ICC). All ICCs were less than 0.05, demonstrating that there were low levels of clustering.

Changes in self-regulatory skills were assessed using paired t-tests for the within-group analysis and ANCOVA, controlling for baseline levels of self-regulation, age, gender and baseline weight, for the between-group analysis. Regression models were used to explore whether baseline data for self-regulation predicted the effect of the intervention on self-regulatory skills changes at 3 month follow-up. The model was adjusted for age, gender and baseline weight and included an interaction term (group condition by predictor).

In the present study we also explored the mediation effects of self-regulatory and automaticity mean changes on the relationship between group condition and weight change at three month follow-up using bootstrapping to estimate indirect effects, and the sobel test. The method used for the mediation analysis was the Baron and Kenny [28], in which the paths of the mediation model are estimated through a series of regression analyses. The sobel test, also called the *product-of-coefficient*, has been widely used in the literature for estimating the indirect effect, but is also considered a conservative method. Since this study tested multiple mediators, Preacher and Hayes [29] recommend also using bootstrapping for testing indirect effects. Bootstrapping is a method that does not impose normality of the sampling distribution and involves multiple resampling of the data set, estimation of the indirect effects and the construct of the confidence interval for the indirect effect [29]. Mean changes in automaticity and in self-regulatory skills over 3 months were tested as mediators, since increases in these constructs were hypothesised to be the underlying mechanisms leading to weight loss. Mediation analyses were adjusted for gender, age, baseline weight, baseline self-regulation mean score and baseline automaticity mean score. Indirect effects were calculated for the total effect, for each mediator and for the contrast of the indirect effects against each other. A 95% Bias Corrected Bootstrapped Confidence Interval was calculated for each indirect effect. Correlations between changes in self-regulation and automaticity in each group condition were also examined.

We performed sensitivity analysis to investigate the potential effect of missing responses for the outcomes using multiple imputations at baseline and follow-up. Multiple imputation models were stratified by study arm and included socio-demographics and self-regulation, automaticity and weight data. A set of 100 imputations were performed.

Finally, descriptive analyses were performed for the data collected from the log books and presented per level of changes in self-regulatory skills, automaticity and weight over three months. Baseline differences were checked between those who sent back the log book and those who did not, using chi-square tests for categorical data and t-tests for continuous data.

## Results

### Participants flow and characteristics

**Fig. 1:**
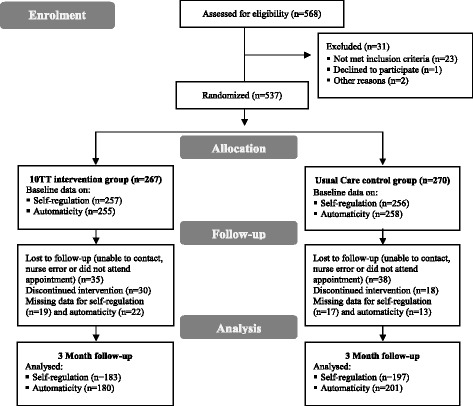
Flow diagram of participation during the study period

Figure 1 displays the flow diagram of the study participation during the first 3-month of the trial. A total of 537 obese patients were eligible to take part in the study; 267 were randomised to the intervention group and 270 to the control group. As shown in Table 1, most of the participants were female (~65%), white (~95%), and approximately half of the participants did not have a degree (~47%). Age, weight, BMI, self-regulation score, and automaticity score at baseline were similar between the two arms. A total of 380 and 381 participants provided data on self-regulation and automaticity at baseline and 3 months (post-intervention), respectively. The non-completers were not significantly different at baseline in socio-demographic characteristics, weight, self-regulation and automaticity outcomes from those who provided data at both time points. The only exception was age, which was significantly greater among completers for self-regulation and automaticity than non-completers.

**Table 1:** Baseline characteristics by randomised group and by completers and non-completers at 3 months for self-regulation and automaticity outcomes

	All participants		Self-regulation outcome		Automaticity outcome	
Characteristics	Intervention group (*N* = 267)	Control group (*N* = 270)	Completers^i^ (N-380)	Non-completers (*N* = 157)	Completers^i^ (*N* = 381)	Non-completers (*N* = 156)
Age (in years)						
Mean (SD)	57.0 (12.8)	57.6 (12.5)	58.5 (11.7)	54.3 (14.4)^**^	58.7 (11.7)	53.9 (14.3)^**^
Gender						
Female, % (n)	66.7 (178)	64.8 (175)	64.5 (245)	68.8 (108)^Δ^	65.1 (248)	67.3 (105)^Δ^
Ethnic group						
White^a^, % (n)	94.7 (252)	95.2 (255)	95.3 (362)	92.9 (145)^Δ^	94.8 (361)	94.2 (146)^Δ^
Other^b^, % (n)	5.3 (15)	4.9 (13)	4.7 (18)	7.1 (11)^Δ^	5.2 (20)	5.8 (9)^Δ^
Qualification						
Non-degree^c^, % (n)	49.6 (129)	44.4 (116)	46.2 (172)	49.0 (73)^Δ^	45.4 (169)	51.0 (76)^Δ^
Degree^d^, % (n)	28.8 (75)	34.9 (91)	33.9 (126)	26.8 (40)^Δ^	33.1 (123)	28.9 (43)^Δ^
Other^e^, % (n)	21.5 (56)	20.7 (54)	19.9 (74)	24.2 (36)^Δ^	21.5 (80)	20.1 (30)^Δ^
Weight^f^ (in kg)						
Mean (SD)	100.4 (17.0)	101.2 (17.5)	100.7 (16.7)	101.3(18.9)^Δ^	100.4 (17.07)	102.06 (18.3)^Δ^
BMI^f^ (in kg/m^2^)						
Mean (SD)	36.1 (4.7)	36.5 (5.4)	36.2 (4.9)	36.5 (5.4)^Δ^	36.2 (5.04)	36.6 (5.2)^Δ^
Self-regulation^g^						
Mean (SD)	2.4(.3)	2.4 (.3)	2.4 (.3)	2.4 (.3)^Δ^	2.4 (.36)	2.4 (.37)^Δ^
Automaticity^h^						
Mean (SD)	4.4 (1.0)	4.5 (.9)	2.4 (.3)	2.4 (.3)^Δ^	4.5 (.9)	4.4 (1.0)^Δ^

**p < .001 ^Δ^ *p* > .05

^a^White British, White Irish or other White background

^b^African, other black background, Indian, Pakistani, Bangladeshi, Chinese, other Asian background, White and Black Caribbean, White and Asian, other mixed background or other ethnic origin

^c^GCSE/School certificate/O-level/CSE, Vocational qualifications or A-level or equivalent

^d^Degree or Post-graduate degree

^e^Still studying, other or do not wish to answer

^f^Data at baseline was available for 536 participants

^g^Self-regulatory skills score ranged from 1 (strongly disagree) to 4 (strongly agree) and data at baseline was available for 513 participants

^h^Automaticity score ranged from 1 (disagree) to 7 (agree) and data at baseline was available for 513 participants

^i^Data at baseline and 3 months

### Post-intervention effect on self-regulatory skills changes

Self-regulatory skills increased significantly over 3 months in both groups (*p* < .001 for all analyses). The between-group analyses showed that at three month follow-up, participants who were given the 10TT intervention had a mean change in self-regulatory skills .08 (95% CI .01; .15, *p* = .019) greater than those who received usual care (Table 2). Sensitivity analysis using multiple imputations gave similar results.

**Table 2:** Effect of the intervention on self-regulation at 3-month follow-up

	Intervention group					Control Group					Mean diff^c^ (95% CI)	*P*
Characteristics	N	Baseline M (SE)	3 M M (SE)	Diff (95% CI)	*P*	N	Baseline M (SE)	3 M M (SE)	Diff (95% CI)	*P*		
Completer analysis												
Self-regulatory skills^a^	183	2.46 (.02)	2.68 (.03)	.22 (.16;.27)	<.001	197	2.49 (.02)	2.62 (.03)	.12 (.08;.17)	<.001	.08 (.01;.15)	.019
Sensitivity analyses^b^												
Self-regulatory skills^a^	267	2.46 (.02)	2.68(.03)	.21 (.16;.26)	<.001	270	2.49 (.02)	2.61 (.03)	.12 (.07;.17)	<.001	.08 (.01;.14)	.012

^a^Self-regulatory skills score ranged from 1 (strongly disagree) to 4 (strongly agree)

^b^Sensitivity analysis using multiple imputation to deal with missing data

^c^Adjusted for gender, age, baseline weight and baseline data for self-regulation

Regression models adjusted for age, gender and baseline weight indicated that lower baseline self-regulatory skills predicted greater changes in self-regulation (B = −.22 SE = .06, *p* < .001) at 3 month follow-up. This result did not differ by arm, as no interaction between baseline data and group condition was found. Analyses using multiple imputations mirrored the results found for completers.

### Mediation analysis

**Fig. 2:**
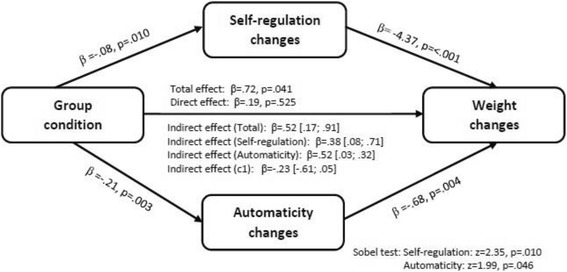
Mediation of self-regulation and automaticity changes on the effect of group condition on weight loss at 3 month

As shown in the preliminary results for this trial, over three months the intervention group lost 1.68 kg (SD 3.20) and the control group 0.84 kg (SD 2.83) and the difference of 0.87 (95% CI -1.47; −.27) was statistically significant [4]. Also, the automaticity of the target behaviours increased significantly more in the 10TT group compared to usual care group condition (adjusted sum difference = 8.45, 95% CI =2.59; 14.32 or adjusted mean changes = .21, 95% CI .07; .35). The present study assessed whether self-regulatory and automaticity mean changes mediated the effect of the intervention on weight changes at three months, when controlling for gender, age, baseline weight, baseline self-regulation and baseline automaticity. The results show the intervention condition significantly predicted self-regulation and automaticity changes, which in turn significantly predicted weight changes at 3 months (Fig. 2). The total effect was significant, while the direct effect was non-significant. Using bootstrapping, the results also indicated that the indirect effects of each mediator, as well as the total effect, were significant. The indirect effect of the contrast of the indirect effects was non-significant. This means that there was no difference in the strength of the indirect effect between the mediators. The sobel test was also assessed and was significant for both mediators: self-regulation changes (z = 2.35, *p* = .010) and automaticity changes (Z = 1.99, *p* = .046). Therefore, changes in both self-regulatory skills and automaticity mediated the effect of the intervention on weight change at 3 month. The sensitivity analysis using multiple imputations showed similar results for both mediators: self-regulation changes (z = 2.54, *p* = .01) and automaticity changes (z = 1.97, *p* = .004).

Regarding the correlation between changes in self-regulatory skills and automaticity, it represented a medium effect in the intervention group (*r* = 0.32 at 3 months) and in the control group (*r* = 0.39 at 3 months). Analyses using multiple imputations provided similar results.

### Descriptive analysis of the 10TT log books

Table 3 presents the descriptive data from the returned 10TT log books. Around 40% of those who received 10TT returned the log books at 3 months. The baseline differences between those who sent back the log book and those who did not were similar to the differences found for completers and non-completers at 3 months. The only exception was for automaticity, which was slightly greater among participants who sent the log book back (M = 4.9 vs M = 4.6, *p* = .041). The majority of participants used the self-monitoring sheets correctly and for a median time of 13 weeks. However, those who showed the greatest changes in self-regulatory skills, automaticity and weight used the self-monitoring sheet for around 14, 14 and 15 weeks, respectively. Most participants recorded their weight for around 13 weeks, but those with the greatest changes in weight recorded it for 15 weeks. In terms of planning, the majority of participants made plans to achieve their behavioural goals for around 10 weeks. However, those with the greatest changes in automaticity did it for 11 weeks. People were expected to manage all 10 target behaviours, however most managed 5 tips per week. People who showed the highest improvement in self-regulatory skills and weight loss managed around 6 tips per week and those with the greatest improvement in automaticity managed 7 tips per week.

**Table 3:** Descriptive data from the log books per level of changes in self-regulatory skills, automaticity and weight over 3 months (Only 10TT participants)

Changes over 3 months	All 10TT participants^a^		10TT participants that returned the Log books^b^		Used self-monitoring sheets		Recorded weight		Made plans		N° times tips were managed ≥5 d/w	Tips managed per week
	N	M (SD)	% (N)	M (SD)	Total % (N)	N° weeks MED	Total % (N)	N° weeks MED	Total % (N)	N° weeks MED	MED	MED
Self-regulation												
Per percentile^c^												
< 75	130	.03 (.22)	38.4 (50)	−.01 (.21)	100 (50)	13	90.0 (45)	13	92.0 (46)	10	76.5	5.5
≥ 75	53	.68 (.27)	35.3 (24)	.67 (.24)	100 (24)	14	91.6 (22)	13	95.8 (23)	10	92.0	6.5
Automaticity												
Per percentile^c^												
< 75	122	.24 (.59)	38.5 (47)	.26 (.47)	95.7 (45)	13	91.5 (43)	13	93.6 (44)	10	70.0	5.5
≥ 75	58	1.54 (.59)	44.8 (26)	1.69 (.74)	100 (26)	14	88.5 (23)	13	92.3 (24)	11	97.5	7.5
Weight												
Per percentile^c^												
< 75	144	−.12 (1.7)	35.4 (51)	−.42 (1.5)	96.1 (49)	13	86.3 (44)	12	92.2 (47)	9	67.0	5.5
≥ 75	56	−5.7 (2.7)	44.6 (25)	−5.7 (3.5)	100 (25)	15	96.0 (24)	15	96.0 (24)	10	90.0	6.5

3 months was equivalent to 15 weeks

*M* mean, *SD* Standard deviation, *MED* Median

^a^All 10TT participants with data for the outcome

^b^All 10TT participants who sent the log books back and had data for the outcomes

^c^Changes to the outcome over 3 months categorised according to the percentile, that is - <75 = medium to low changes and ≥75 = greater changes

## Discussion

This study is the first to explicitly assess the potential mechanisms of a brief habit-based intervention for weight loss in a population-based sample of obese adults. The study showed that 10TT promoted changes in self-regulatory skills and that these changes, alongside changes in automaticity, mediated the effect of 10TT on weight loss. Furthermore, participants who engaged more with the intervention in terms of number of weeks monitoring the target behaviours, recording weight, and making plans, experienced the greatest changes in self-regulatory skills, automaticity and weight.

The results of this study are in line with the suggestion that habit-based interventions help people to improve their self-regulatory capacity, since they require people to make goals, plan and monitor their behaviour [9, 14]. This is comparable to other intervention studies that have applied planning techniques, in which changes in self-regulatory skills were found over a short [30, 31] and long period of time [32]. However, this is the first study to show that a planning technique within a habit intervention improves self-regulatory skills. Importantly, the 10TT intervention was particularly effective at promoting self-regulatory skills among those patients who had poor self-regulation skills at baseline, although ceiling effects may have affected this result. However, due to the nature of 10TT (a brief, self-help intervention), the specificity and relevance of the goals and plans formed by the participants is not known. The quality of plans is relevant for habit formation, as they need to be context specific in order to make the target behaviour become more automatic. Future studies should assess the quality of plans made by participants and explore how this relates to habit formation.

According to the previous results of this trial, patients who received the 10TT also experienced greater increases in automaticity than those who received usual care at 3 months [4]. The present study demonstrated that changes in both self-regulatory skills and automaticity mediated the effect of 10TT on weight loss at 3 months. Given that the majority of studies do not assess the mechanism for an intervention’s success [32], this is an important finding which suggests the intervention works as it is intended to. This is also in line with recent evidence suggesting that nutrition and weight loss interventions that include self-regulation components tend to be more effective [33, 34]. The observed correlation between changes in automaticity and self-regulatory skills also adds to our understanding of the habit-formation process, as it suggests that self-regulatory skills may be required for the development of automaticity [35, 36]. Self-regulatory skills are thought to help individuals to act according to an intended behaviour [9, 14] and may also help to both suppress impulse tendencies toward temptations [15] and prevent the loss of healthy habits when environmental cues change [10].

The log book data showed that participants who monitored their weight and target behaviours more frequently, and who made more plans, showed the greatest improvement in self-regulatory skills and automaticity, and also experienced the greatest weight loss. This is an indication that the intervention worked best when engaged with and adhered to. Although the difference in self-monitoring between those who showed the greatest and lowest engagement was only around 1 to 2 weeks, this fits with habit theory. According to Lally and Gardner [9] habit formation may vary widely and habits can be formed over as little as 18 days as many as 254 days, depending on the complexity of the behaviour, and as other aspects. Furthermore, a study conducted by Lange, Richert, Koring, Knoll, Schwarzer and Lippke [31] showed that significant improvements in self-regulation can be observed over just a week. Future studies should explore ways to improve engagement and adherence, for example the use of novel technologies to facilitate self-monitoring. In addition, the 10TT intervention only addresses forming habits and does not include a self-regulatory training component to help participants break existing habits. This could be important because breaking habits require more effortful self-regulatory skills in order to disrupt cue-response associations [9]. Future studies should explore the effect of adding self-regulatory training specifically focused on breaking habits to the current advice on forming habits. This could potentially enhance the effects of the 10TT intervention.

A strength of this study was that the intervention was delivered by health professionals from primary care across England, which provides direct evidence for its effectiveness in clinical practice. However, there are limitations concerning the generalisability of the results, which were presented with the previous findings from this trial [4]. Briefly, participants were not blinded to their condition, although the 3-month follow-up assessment was done by a health professional blinded to condition allocation. Ethnic minorities and men were under-represented and the sample was slightly older compared with the population of adults with obesity described in the Health Survey of England.

There are also limitations related specifically to the current analyses. The results for changes in self-regulation and automaticity should be interpreted as exploratory, as the trial was only powered to detect differences in weight change between the group conditions. Self-regulation was measured using an adapted version of the SRQ, which excluded the middle response option from the original version to encourage people to commit one way or the other, but as a consequence may have increased the risk of ceiling effects. Future studies should aim to replicate these analyses using a valid and reliable measure of eating self-regulatory skills, such as the recently developed Self-Regulation Questionnaire of Eating Behaviour (SREBQ) [37]. The automaticity of the target behaviours was assessed using only one item from the 12-item Self-report Habit Index [27], and may not be comprehensive enough to assess habit formation. Future studies should consider using the shortened 4-item version of this questionnaire, which has been recently validated [38]. In this study, automaticity also represented the overall score for the automaticity of the target behaviours. As a consequence, it was not possible to draw conclusions about which behaviours had become habitual and it may have overestimated the effect of the intervention. Future studies should explore changes in each of the eating, activity and self-weighing behaviours and their automaticity separately using valid and reliable measures, as opposed to single items.

Furthermore, given the measures used in this study were all self-report, changes in self-regulatory skills and automaticity may represent the individuals perception of change, rather than actual change. Objective and technologic-based methods to assess nutrition, physical activity and healthy behaviours could promote more accurate data on these behaviours [39]. Other aspects may have also played a role on the effect of the intervention on weight loss that were not included in these analyses. For example, social support has also been identified as an important aspect of behaviour change [40], and this should be further explored. In addition, the analysis of the pathways was missing data for the variables of interest (compromising internal validity) and of course nullifying randomisation so that the results are more in keeping with that of a cohort study analysis rather than a RCT. Finally, qualitative analysis on the specificity of plans made by the participants and their relationship with habit formation could also further the understanding of the effect of this intervention.

## Conclusions

In conclusion, our findings suggest that a habit-based intervention can enhance self-regulatory skills, especially among people with lower levels of self-regulatory skills at baseline. Furthermore, changes in self-regulation and automaticity are the underlying mechanisms by which 10TT promoted weight loss in adults with obesity, supporting the theoretical basis of the intervention. This study also provided evidence that greater engagement with the intervention was associated with greater improvements in self-regulatory skills, automaticity and weight loss. Future studies should explore whether the effect of the 10TT intervention on self-regulation and automaticity can be enhanced through facilitating engagement with the log books (e.g. through digital self-monitoring) and the effect of adding self-regulatory training for breaking existing habits on weight loss.

## Additional files

Additional file 1: CONSORT 2010 checklist of information to include when reporting a randomised trial* (DOC 218 kb)

Additional file 2: Table S1. 10 Target eating and activity behaviours plus self-weighing and the 16 automaticity questions (DOCX 13 kb)

Additional file 3: Table S2. Descriptive data of the automaticity of each target behaviour (DOCX 18 kb)

## Abbreviations

10TT: 10 Top Tips intervention
BMI: Body Mass Index
SREBQ: Self-Regulation of Eating Behaviour Questionnaire
SRQ: Self-Regulation Questionnaire
