# Supplementary material for: The role of self-regulatory skills and automaticity on the effectiveness of a brief weight loss habit-based intervention: secondary analysis of the 10 top tips randomised trial
Source: Int J Behav Nutr Phys Act. 2017 Sep 5;14:119. doi: 10.1186/s12966-017-0578-8 (PMC5583960; doi:10.1186/s12966-017-0578-8)
Supplement: Supplementary file 2 — 10 Target eating and activity behaviours plus self-weighing and the 16 automaticity questions (DOCX 13 kb) [file 12966_2017_578_MOESM2_ESM.docx]

Additional file 2: **Table S1** 10 Target eating and activity behaviours plus self-weighing and the 16 automaticity questions

| **Target Behaviour** | **Question** | **Response options** |
| --- | --- | --- |
| 1. Keep to your meal Routine | 1. Eating my meals around the same time every day is something I do automatically | 7-point Likert scale from ‘none of the time’ to ‘all of the time. |
| 2. Go reduced fat | 2. Choosing reduced fat foods is something I do automatically  3. Using high fat foods only sparingly is something I do automatically |  |
| 3. Walk off the weight | 4. Walking 10 000 steps a day is something I do automatically |  |
| 4. Pack a healthy snack | 5. Choosing healthy rather than unhealthy snacks is something I do automatically |  |
| 5. Look at the labels | 6. Reading the labels when buying food is something I do automatically  7. Reading the labels when preparing food is something I do automatically |  |
| 6. Caution with your portions | 8. Avoiding large portions (except of fruit and vegetables) is something I do automatically  9. Avoiding second helpings is something I do automatically |  |
| 7. Up on your feet | 10. Standing for at least ten minutes every hour is something I do automatically |  |
| 8. Think about your drinks | 11. Drinking water and sugar-free drinks instead of other soft drinks is something I do automatically  12. Drinking more than two units of alcohol a day is something I do automatically |  |
| 9. Focus on your food | 13. Eating at a table is something I do automatically  14. Eating in front of the TV is something I do automatically |  |
| 10. Don’t forget your 5-a-day | 15. Eating at least five portions of fruit and vegetables a day is something I do automatically |  |
| Self-weighing every day | 16. Weighing myself is something I do automatically |  |
