# Supplementary material for: The role of self-regulatory skills and automaticity on the effectiveness of a brief weight loss habit-based intervention: secondary analysis of the 10 top tips randomised trial
Source: Int J Behav Nutr Phys Act. 2017 Sep 5;14:119. doi: 10.1186/s12966-017-0578-8 (PMC5583960; doi:10.1186/s12966-017-0578-8)
Supplement: Supplementary file 3 — Descriptive data of the automaticity of each target behaviour (DOCX 18 kb) [file 12966_2017_578_MOESM3_ESM.docx]

Additional file 3**: Table S2** Descriptive data of the automaticity of each target behaviour

| **Automaticity of Target behaviour** | **Intervention group** | | | | |  | **Control group** | | | | |
| --- | --- | --- | --- | --- | --- | --- | --- | --- | --- | --- | --- |
|  | **Baseline** | | **3 months** | | **Changes** |  | **Baseline** | | **3 months** | | **Changes** |
|  | N | M(SD) | N | M(SD) | M(SD) |  | N | M(SD) | N | M(SD) | M(SD) |
| 1. Keep to your meal routine | 252 | 5.1 (1.9) | 188 | 5.6 (1.5) | .27 (.79) |  | 256 | 5.3 (1.8) | 207 | 5.6(1.3) | .01 (.75) |
| 2. Go reduced fat | 254 | 4.3 (1.6) | 207 | 5.1 (1.4) | .76 (1.6) |  | 256 | 4.5 (1.6) | 207 | 5.0 (1.4) | .37 (1.5) |
| 3. Walk off the weight | 256 | 4.6 (2.1) | 189 | 5.4 (1.6) | .10 (1.5) |  | 260 | 4.7 (2.0) | 205 | 4.9 (1.7) | .12 (1.4) |
| 4. Pack a healthy snack | 253 | 4.0 (1.7) | 184 | 5.0 (1.5) | .96 (1.8) |  | 256 | 4.3 (1.7) | 203 | 5.2 (1.4) | .73 (1.8) |
| 5. Look at the labels | 253 | 3.6 (1.7) | 186 | 4.7 (1.7) | .71 (1.1) |  | 256 | 3.8 (1.8) | 202 | 4.5 (1.6) | .38 (.99) |
| 6. Caution with your portions | 256 | 4.7 (1.6) | 189 | 5.5 (1.3) | .53 (.84) |  | 259 | 4.8 (1.6) | 207 | 5.4 (1.3) | .42 (.92) |
| 7. Up on your feet | 254 | 3.3 (2.1) | 185 | 3.6 (2.0) | .28 (1.0) |  | 256 | 3.4 (2.1) | 207 | 3.8 (2.0) | .25 (1.1) |
| 8. Think about your drinks | 248 | 5.7 (1.3) | 185 | 6.0 (1.1) | .23 (.74) |  | 248 | 5.7 (1.3) | 201 | 5.9 (1.2) | .26 (.74) |
| 9. Focus on your food | 255 | 4.6 (1.9) | 189 | 5.2 (1.7) | .17 (.67) |  | 259 | 4.6 (1.9) | 208 | 4.8 (1.8) | .10 (.69) |
| 10. Don’t forget your 5-a-day | 258 | 4.8 (2.0) | 190 | 5.5 (1.6) | .44 (.98) |  | 256 | 4.8 (1.9) | 209 | 5.3 (1.7) | .16 (.86) |
| Record your weight | 260 | 3.4 (2.4) | 187 | 4.6 (2.2) | 1.1 (2.1) |  | 258 | 3.3 (2.3) | 208 | 3.9 (2.2) | .29 (1.1) |
| Overall | 250 | 4.6 (1.0) | 184 | 5.3 (1.0) | .66 (.83) |  | 255 | 4.7 (.9) | 204 | 5.2 (.8) | .45 (.80) |
